# MOF‐Based Nanotubes to Hollow Nanospheres through Protein‐Induced Soft‐Templating Pathways

**DOI:** 10.1002/advs.201801684

**Published:** 2019-01-11

**Authors:** Yingjie Du, Jing Gao, Liya Zhou, Li Ma, Ying He, Xuefang Zheng, Zhihong Huang, Yanjun Jiang

**Affiliations:** ^1^ School of Chemical Engineering Hebei University of Technology No. 8 Guangrong Road Hongqiao District Tianjin 300130 China

**Keywords:** hollow composites, hydrogels, metal–organic frameworks, protein‐induced, sodium deoxycholate

## Abstract

A controllable and facile strategy is established for constructing metal–organic frameworks‐based (MOF‐based) hollow composites via a protein‐induced soft‐templating pathway. Using metal‐sodium deoxycholate hydrogel as soft‐template, nanotubes are gained while the protein is absent. With the presence of protein, hollow nanospheres structure are prepared by changing the amount of protein. To verify the universality of the proposed pathway, two kinds of proteins (*Burkholderia cepacia* lipase and penicillin G acylase) and three kinds of MOF (ZIF‐8, ZIF‐67, and Fe‐MOF) are adopted as model proteins and materials, and the obtained protein‐containing composites (named protein@H‐MOF) possess high bioactivity and stability. This proposed strategy provides a facile method for preparing MOF‐based composites under mild conditions, facilitating the applications of MOF in the fields of biocatalyst construction, biomolecule encapsulation, and drug delivery.

Due to their diverse fascinating topologies and properties, metal–organic frameworks (MOFs) have attracted tremendous interest in the fields of gas adsorption, drug delivery, catalysis, and enzyme immobilization.^1^ Over the past few years, various types of nanobiocatalysts have successfully been prepared via various methods by using different MOF matrices as supports.[[qv: 1e,2]] In recent years, both capsules and hollow sphere materials have attracted increasing interest as supports to produce nanocomposites because they can provide a favorable microenvironment for biomolecules.^3^ The hollow cavity can accommodate a high loading amount of biomolecule cargoes, and the thin capsule wall endows the resulting nanocomposites with the rapid mass transfer, low cargo leaching, and high activity recovery.[[qv: 3a,d]] Sodium deoxycholate (NaDC) is a water‐soluble bile salt which is very important in biology and medicine as a biocompatible surfactant for purifying DNA and proteins.^4^ NaDC hydrogel can maintain the high activity of biomolecules.^4^ The metal‐NaDC hydrogel is also a suitable template for hollow material synthesis because of the tunable size and shape of the hydrogel.[[qv: 4b,5]] The formation of the metal‐NaDC hydrogel is influenced by various conditions including ion concentration, pH, and other conditions.[[qv: 4a,6]] In addition, the high biocompatibility of the metal‐NaDC hydrogel can also protect biomolecules from an extreme nonphysiological environment.[[qv: 4a,6,7]]

Thus, herein, a metal‐NaDC hydrogel was adopted as the soft template for constructing hollow MOF‐based composites in a facile approach for the first time. As a subfamily of MOFs, zeolitic imidazolate frameworks (ZIFs) are porous crystalline materials with zeolite framework structures formed by coordination between transition metal ions and imidazole derivative ligands.[[qv: 1b,e]] ZIF‐8 was chosen as the model material for the hollow MOF‐based composite construction because of its high surface area and remarkably high chemical and thermal stability.[[qv: 1a,2c]]*Burkholderia cepacia* lipase (BCL, EC 3.1.1.3), a type of hydrolase, has been widely used in hydrolysis, esterification, and transesterification reactions and was chosen as the model protein in this study.^8^

The effects of the reaction conditions on the morphology of Zn^2+^‐NaDC hydrogel fibers and the subsequent construction of the hollow ZIF‐8‐based composite (H‐ZIF‐8) were investigated first. The glazed Zn^2+^‐NaDC hydrogel fibers can be constituted through the self‐aggregation of Zn^2+^ ions and NaDC at pH 7 in the absence of BCL (Figure S1a, Supporting Information). Figure S1b–f (Supporting Information) shows the surface morphology of Zn^2+^‐NaDC hydrogel fibers at various BCL concentrations, and it can be seen that the surface of Zn^2+^‐NaDC hydrogel fibers becomes increasingly rougher as the BCL protein concentration increases. After the hydrogel was formed, 2‐methylimidazole (2‐MeIM) was added as the ligand for ZIF‐8. The results of rheological properties (Figure S1g,h, Supporting Information) showed that the hydrogel exhibited more and more solid‐like rheological behavior with the protein amount increasing, which are consistent with the scanning electron microscopy (SEM) images. This phenomenon can be ascribed to the multiple carboxylate groups structure of the protein that influences the interactions between NaDC and metal ions.[[qv: 5d,6]] Moreover, interactions between metal ions and carboxylate groups of protein molecules might lead to aggregation of the metal ions and proteins, which results in defect sites of hydrogel fibers. The ZIF‐8‐based nanotube was constructed in the absence of protein (**Figure** **1**a and Figure S2a, Supporting Information), and limited H‐ZIF‐8 spheres were formed gradually when the concentration of BCL was increased from 0 to 2 mg mL^−1^ (Figure 1b,c). The Zn^2+^‐NaDC hydrogel fibers containing protein decomposed into nanospheres within 2 h of reaction, generating a soft template for H‐ZIF‐8 nanosphere formation.[[qv: 6c]] Thus, spherical BCL@H‐ZIF‐8 was formed when the protein concentration was greater than 2.7 mg mL^−1^. As shown in Figure 1 and Figure S2 (Supporting Information), the transmission electron microscopy (TEM) results confirmed the hollow morphology of the H‐ZIF‐8 composites, verifying that a high protein concentration might be beneficial for H‐ZIF‐8 composite construction.

**Figure 1:**
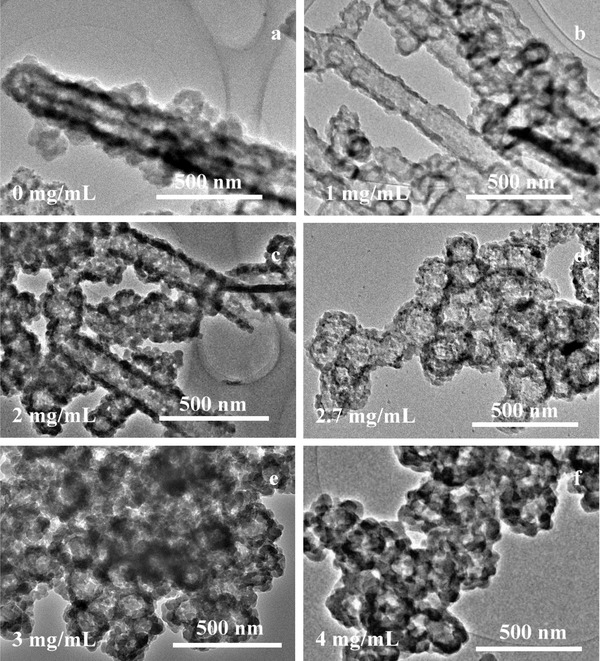
TEM images of BCL@H‐ZIF‐8 constructed with various protein concentrations.

This phenomenon was observed because the Zn^2+^‐NaDC hydrogel fibers is so pyknotic under dilute protein solution conditions that 2‐MeIM could not break the hydrogel fibers.[[qv: 4a,6c]] Under high protein concentrations, protein molecules were embedded in the Zn^2+^‐NaDC hydrogel fibers, decreasing the density of the fibers and making the hydrogel unstable enough for 2‐MeIM to remove the zinc ions from the hydrogel.[[qv: 6c,7b]] It should be noted that the effect of the order of addition of the precursors on the construction of H‐ZIF‐8 was also investigated in preliminary experiments. As shown in Figure S3 (Supporting Information), the sequence of addition of Zn^2+^ and BCL were exchanged in group 2, and the 2‐MeIM was added at first in group 3. Although the solution turned milky white when the Zn^2+^ solution was added in all three groups, only the order of addition of group 1 (the adopted approach) led to the successful formation of H‐ZIF‐8.

Next, the impact of the reaction pH on the hollow composite construction was investigated. As seen in Figure S4a–d (Supporting Information), ZIF‐8 crystals formed at pH 5 and 6 (the pKa of NaDC is 6.8), while hollow BCL@H‐ZIF‐8 appeared as the pH was increased from 7 to 8 (above the pKa of NaDC). These results indicate that pH is an important factor because the pH environment can influence ionic interactions during hydrogel formation.[[qv: 6a]] Note that the BCL@H‐ZIF‐8 composite nanospheres can form at pH 7 and 8 with the appropriate protein concentration (Figure 1, Figures S1 and S5a, Supporting Information).

The effect of Zn^2+^ concentration was another important factor in ionic interactions and also investigated over the range of 0.075 to 0.6 × 10^−3^ m (**Figure** **2**). As seen in Figure 2a,b, the BCL@H‐ZIF‐8 composite spheres did not form at Zn^2+^ concentrations below 0.15 × 10^−3^ m, possibly because excessively low concentrations of metal ions have adverse effects on the formation of both the Zn^2+^‐NaDC hydrogel and ZIF‐8.[[qv: 6b]] The SEM and TEM results (Figure 1c, Figure S2c, Supporting Information, and Figure 2, Figure S6, Supporting Information) suggested that 0.3 × 10^−3^ m Zn^2+^ was the appropriate concentration for the formation of H‐ZIF‐8 with the ideal morphology.

**Figure 2:**
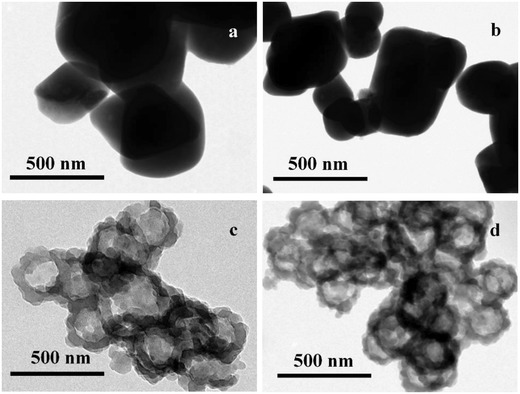
TEM images of BCL@H‐ZIF‐8 constructed under various Zn^2+^ concentrations.

The X‐ray diffraction (XRD) pattern of the BCL@H‐ZIF‐8 contains the major crystallographic planes of ZIF‐8, such as peaks at 7.5°, 10.5°, and 12.5° (Figure S5b, Supporting Information), demonstrating the successful formation of ZIF‐8 crystals on BCL@H‐ZIF‐8, which is in agreement with previous reports.[[qv: 1b,9]] The XRD patterns of the obtained composites constructed under various Zn^2+^ concentrations and pH values are given in Figure S5c,d (Supporting Information). It is worth noting that there are some different peaks appearing in the XRD patterns of various BCL@H‐ZIF‐8. From these results, it can be seen that the protein concentration, pH, and Zn^2+^ concentration cannot only influence the morphology but also change the crystal form of the obtained composites.[[qv: 9b,c]] Thus, by considering the results of XRD data, SEM, and TEM, it can be concluded that the well‐defined hollow BCL@H‐ZIF‐8 nanospheres can be prepared only under appropriate conditions.

On the basis of the above results, the formation mechanism of the MOF‐based composites is proposed and described in **Scheme** **1**. The hydroxy and carboxyl groups give the NaDC molecules planar polarity and can offer the adequate negative charge, which is essential for metal ions‐NaDC aggregation in aqueous solution.[[qv: 4,5,6d,e]] As shown in Scheme 1a, MOF‐based nanotubes rather than hollow spheres will form around the hydrogel fiber template when the protein is absent. As shown in Scheme 1b, the protein molecules are first aggregated into the soft metal ion‐surfactant nanofiber hydrogel. Zn^2+^ ions might aggregate not only with NaDC molecules but also protein molecules because protein molecules also have carboxyl groups as same as NaDC molecules.[[qv: 4b,6c,e]] This might influence the compactness and roughness of the hydrogel fiber by gradually increasing the formation of defect sites as the protein concentration increases (Figure S1, Supporting Information).[[qv: 6c]] As a result, Zn^2+^ ions are more readily captured from the hydrogel by 2‐MeIM. In other words, because of the presence of protein molecules in the Zn^2+^‐NaDC hydrogel fibers, Zn^2+^ ions in the solution and Zn^2+^‐NaDC hydrogel fibers can be captured by 2‐MeIM simultaneously. When the Zn^2+^ ions around the defect sites were captured by 2‐MeIM, the hydrogel fibers may be gradually decomposed into nanospheres. And the hydrogel nanospheres can act as soft templates for the synthesis of the H‐ZIF‐8 composites. Furthermore, with Zn^2+^ irons being taken away by 2‐MeIM, the Zn^2+^‐NaDC hydrogel fibers will be decomposed and removed easily during the washing process.[[qv: 6c]] In addition, the alkaline 2‐MeIM solution can change the pH of the reaction system, which might influence the morphology of the hydrogel fibers and further influence the formation of the H‐ZIF‐8 composites under appropriate protein concentration.^6^ Thus, a novel protein‐induced approach controllably construct MOF‐based hollow composites was established.

**Scheme 1:**
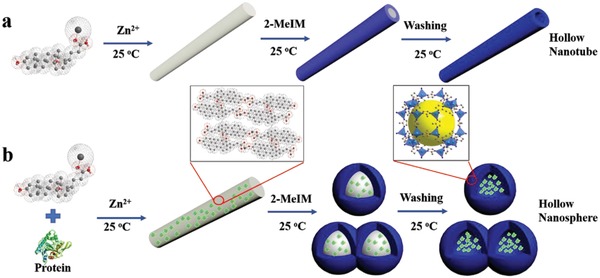
a,b) Illustration of the procedure for synthesizing hollow MOF tubes and the protein‐induced hollow MOF‐based composite spheres.

Furthermore, the result of X‐ray photoelectron spectroscopy (XPS) (Figure S7a, Supporting Information) indicated the existence of a bond between the zinc ion and nitrogen.^10^ The band at 421 cm^−1^ of Fourier transform infrared (FTIR) spectra (Figure S7b, Supporting Information) can be assigned to Zn—N bond stretch, which also confirmed the successful formation of the ZIF‐8 framework structure.[[qv: 9b,c]] The pore size distribution of BCL@H‐ZIF‐8 confirmed the presence of micropores (1.580 nm) and mesopores (3.403 nm) in the BCL@H‐ZIF‐8, verifying that the microporous structure of ZIF‐8 was maintained while the mesopores appearing in the shell of the hollow nanospheres. In general, the presence of mesopores can offer larger pore volume and may reduce the Brunauer–Emmett–Teller (BET) surface area of the microporous materials.^11^ Compared to pure ZIF‐8 (1012 m^2^ g^−1^, Figure S7d, Supporting Information), the BET surface area of BCL@H‐ZIF‐8 (328.3 m^2^ g^−1^, Figure S7c, Supporting Information) decreased, which can be due to the presence of mesopores (3.403 nm) in the shell.[[qv: 2c,3c,11]] TEM mapping (**Figure** **3**a,b) and TEM line scanning (Figure 3c,d) showed that the Zn element was homogeneously distributed on the shell, which confirmed the hollow structure of BCL@H‐ZIF‐8.^12^ Considering that biomolecules always require neutral pH conditions, in subsequent experiments, BCL@H‐ZIF‐8 was constructed under the following conditions: 2.7 mg mL^−1^ protein, 0.3 × 10^−3^ m Zn^2+^, and pH 7.

**Figure 3:**
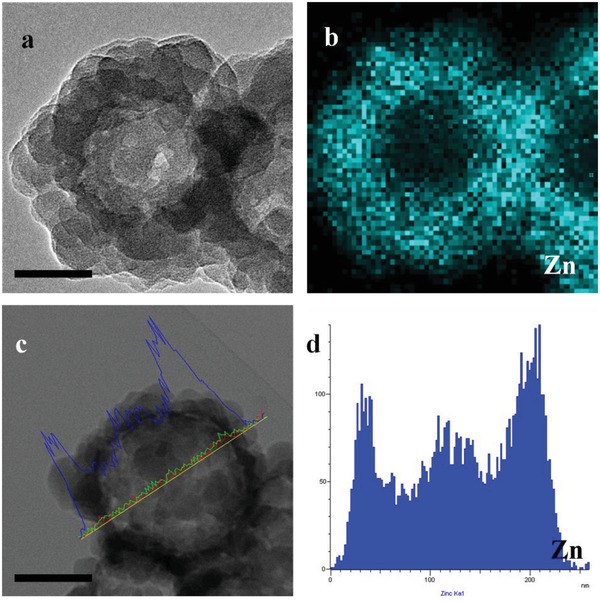
a,c) STEM images (inset scale bars are 100 nm), b,d) elemental mapping and element line scan images of Zn on BCL@H‐ZIF‐8.

To investigate the performance of BCL@H‐ZIF‐8 and verify that the metal‐NaDC hydrogel can protect the BCL molecules, the activity and stability of the BCL@H‐ZIF‐8 were investigated. The protein was almost encapsulated in the composites because there was no protein that can be detected in the supernatant during the composites construction process. As shown in Tables S1 and S2 (Supporting Information), the obtained BCL@H‐ZIF‐8 possessed a substantially higher activity recovery (69.58%) than the composite formed from BCL embedded in a pure ZIF‐8 crystal (BCL@ZIF‐8, 8.749%). The high activity recovery might be due to the following reasons: (1) the Zn^2+^‐NaDC hydrogel can offer a comfortable microenvironment for BCL during the H‐ZIF‐8 construction process; (2) the porous thin wall of H‐ZIF‐8 can decrease the mass transfer resistance and can protect BCL molecules from external biotoxic molecules; and (3) the free state of the BCL molecules in the cavity can facilitate the interaction between BCL and the substrate molecules.[[qv: 2b,c]]

Generally, immobilization always results in an order of magnitude the change in the kinetic parameters compared to those of free enzymes.[[qv: 3c,13]] As seen in **Table** **1**, compared to free BCL, BCL@ZIF‐8 exhibited a sharp increase in *K*_m_ (Michaelis–Menten constant) and decreases in both *K*_cat_ (turnover number) and *K*_cat_/*K*_m_. Additionally, the slight decrease in the *K*_cat_ value of BCL@H‐ZIF‐8 indicated a reduction in the affinity between BCL and the substrate molecules.[[qv: 13a]] The increase in the apparent *K*_m_ and the decrease in *K*_cat_/*K*_m_ may be due to the mass transport resistance for substrates or products into and out of the hollow nanospheres.^14^ Furthermore, the slight change in *K*_m_ and *K*_cat_ also indicated that the conformation and structure of the BCL molecules were protected during the BCL@H‐ZIF‐8 construction process.[[qv: 2c,3c]] Hence, the kinetic parameters and activity recovery results indicated that the proposed approach for preparing BCL@H‐ZIF‐8 can offer better protection for proteins during composite construction than biomimetic mineralization process approach for preparing ZIF‐8 immobilized enzymes (BCL@ZIF‐8).

**Table 1:** Kinetic parameters of free BCL, BCL@ZIF‐8, and BCL@H‐ZIF‐8

	Free BCL	BCL@ZIF‐8	BCL@H‐ZIF‐8
*K*_m_ (×10^−3^ m)	1.800	14.75	4.228
*K*_cat_ (S^−1^ × 10^4^)	2.040	0.07418	0.2967
*K*_cat_/*K*_m_ (S^−1^ mM^−1^ × 10^4^)	1.133	0.005029	0.07018

In addition, as seen in Table S1 and Figure S8 (Supporting Information), the observed rate constant (*K*_obs_) value of BCL@H‐ZIF‐8 (0.01029 s^−1^) was slightly less than that of free BCL (0.01513 s^−1^). The Kobs results indicated that the biocompatible Zn^2+^‐NaDC hydrogel can minimize protein conformational changes while maintaining more activity.^14^ Furthermore, as shown in Figure S9 (Supporting Information), free BCL and BCL@H‐ZIF‐8 were incubated with papain, trypsin, and alkaline protease for 3 h each. BCL@H‐ZIF‐8 retained more activity than free BCL, which indicated that the H‐ZIF‐8 shell can protect BCL from proteolysis by preventing the proteases from entering the spheres.[[qv: 3c,15]]

Polar organic solvents, such as acetonitrile, usually decrease enzyme activity sharply because they are denaturants for proteins,^16^ but acetonitrile is inevitably usually used as a solvent for enzyme industrial catalysis.^17^ As shown in **Figure** **4**a, to verify the enzyme tolerance in acetonitrile, free BCL and BCL@H‐ZIF‐8 were incubated in a 40% (v/v) acetonitrile solution for a specific time. Free BCL and BCL@H‐ZIF‐8 maintained 70.23 and 98.62% of their initial activity during the initial 10 min of incubation. After 130 min of incubation, BCL@H‐ZIF‐8 retained over 60% of its initial activity, while free BCL only retained ≈45% of its initial activity. Compared to free BCL, BCL@H‐ZIF‐8 had a higher tolerance, which is attributed to the H‐ZIF‐8 shell decreasing the interaction between enzyme molecules and acetonitrile while also maintaining the necessary water around the protein molecules.^16^, ^17^ Reuse of free enzymes is usually difficult due to their mutability and the high cost of purification technology.^18^ After recycling nine times, the BCL@H‐ZIF‐8 retained over 50% of its initial activity because the H‐ZIF‐8 shell prevented the leakage and denaturation of the BCL molecules (Figure 4b).[[qv: 11a,19]]

**Figure 4:**
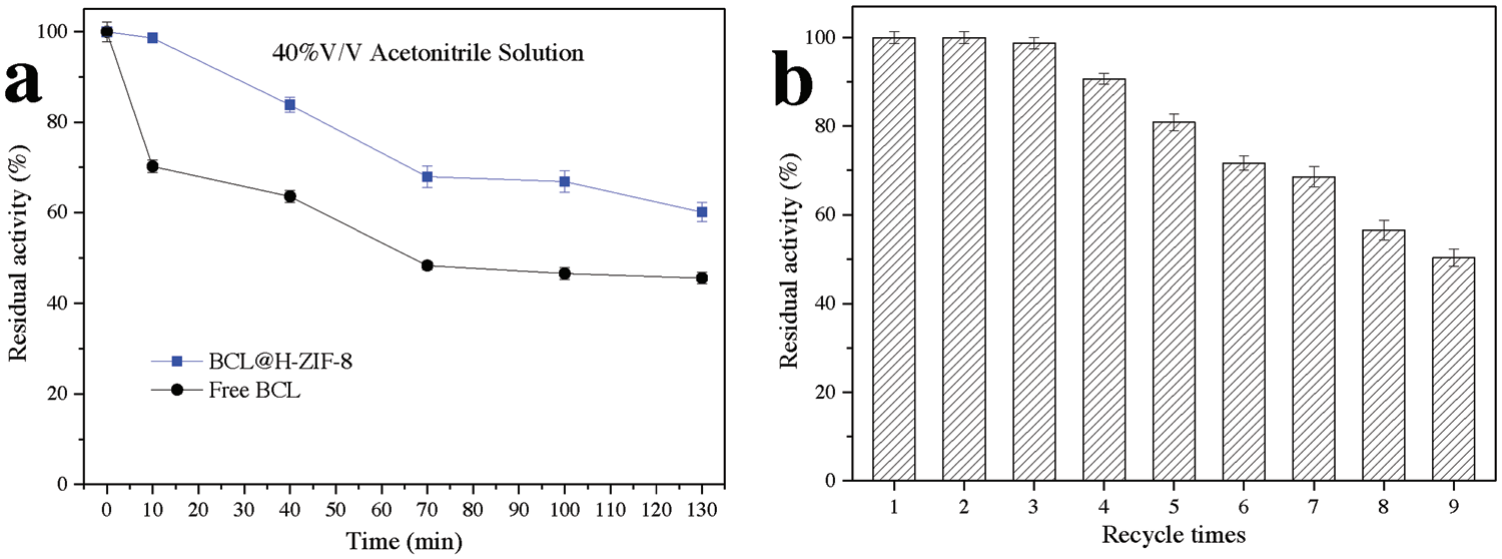
a) Stability of free and immobilized BCL against 40% v/v acetonitrile solution and b) reusability of BCL@H‐ZIF‐8.

To demonstrate the versatility of this novel approach, ZIF‐67 and Fe‐MOF were adopted as another model MOFs to construct ZIF‐67‐based hollow composite (BCL@H‐ZIF‐67, Figure S10a, Supporting Information) and Fe‐MOF‐based hollow composite (BCL@H‐Fe‐MOF, Figure S10b, Supporting Information). These two types of hollow spheres were also constructed successfully with satisfactory morphology. Furthermore, the activity of BCL in BCL@H‐ZIF‐67 and BCL@H‐Fe‐MOF can be maintained (Table S2, Supporting Information). The successful construction of these protein‐contained composites indicated that the proposed approach can be extended to different kinds of MOFs. Furthermore, this developed approach was also applied to construct another kind of enzyme‐containing composite. Penicillin G acylase (PGA, EC 3.5.1.11), which has been extensively used in many valuable biocatalytic reactions, was chosen as the model enzyme.^20^ PGA‐containing hollow MOF‐based composite spheres (PGA@H‐ZIF‐8, Figure S8c, Supporting Information) were also constructed successfully with high activity recovery (Table S2, Supporting Information).

In summary, a novel and general approach for the controllable construction of protein‐containing hollow MOF composites has been established successfully under mild conditions. MOF‐based nanotubes and hollow nanospheres can be obtained controllably by tuning this protein‐induced process. The biocompatibility of this approach endows hollow MOF composites with a broad range of applications in biocatalyst construction, biomolecule encapsulation, drug delivery, and controlled release.

## Conflict of Interest

The authors declare no conflict of interest.

## Supporting information

Supplementary
