# Supplementary material for: MOF‐Based Nanotubes to Hollow Nanospheres through Protein‐Induced Soft‐Templating Pathways
Source: Adv Sci (Weinh). 2019 Jan 11;6(6):1801684. doi: 10.1002/advs.201801684 (PMC6425429; doi:10.1002/advs.201801684)
Supplement: Supplementary file 1 — Supplementary [file ADVS-6-1801684-s001.pdf]

Copyright WILEY-VCH Verlag GmbH & Co. KGaA, 69469 Weinheim, Germany, 2019.

ADVANCED SCIENCE
Open Access

Supporting Information

for *Adv. Sci.,* DOI: 10.1002/advs.201801684

MOF-Based Nanotubes to Hollow Nanospheres through Protein-Induced Soft-Templating Pathways

Yingjie Du, Jing Gao, Liya Zhou, Li Ma, Ying He, Xuefang Zheng, Zhihong Huang, and Yanjun Jiang**

Copyright WILEY-VCH Verlag GmbH & Co. KGaA, 69469 Weinheim, Germany, 2016.

Supporting Information

MOF-based Nanotubes to Hollow Nanospheres through Protein-induced Soft-templating Pathways

Yingjie Du, Jing Gao, Liya Zhou, Li Ma, Ying He, Xuefang Zheng, Zhihong Huang, Yanjun Jiang**

Experimental

Reagents and chemicals:

Burkholderia cepacia lipase (BCL; specific activity: 3685 $U \cdot g^{-1}$ protein) and penicillin G acylase (PGA; specific activity: 41460 $U \cdot g^{-1}$ protein) were purchased from Beijing Ding Guo Bioengineering Co. Ltd., China. 2-Methylimidazole (2-MeIM), sodium deoxycholate (NaDC, 99.9%) and fumaric acid (FA) were purchased from Sigma Aldrich (Shanghai) Trading Co. Ltd., China. Zinc nitrate hexahydrate ($Zn(NO_3)_2 \cdot 6H_2O$, 99.99%), iron(III) chloride hexahydrate ($Fe(Cl)_3$ $6H_2O$, 99%) and cobaltous nitrate hexahydrate ($Co(NO_3)_2 \cdot 6H_2O$, 99.99%) were purchased from Aladdin Industrial Corporation, Shanghai, China. Disodium hydrogen phosphate (Na_2HPO_4), sodium dihydrogen phosphate (NaH_2PO_4), methanol, tris(hydroxymethyl)aminomethane (Tris), hydrochloric acid (HCl, 37.5%) and acetonitrile were purchased from Tianjin Chemical Reagent Company. Penicillin G potassium salt (PGAK) and 4-dimethylaminobenzaldehyde (PDAB) were purchased from Tianjin Guangfu Jingxi Chemical Company. 6-Aminopenicillanic acid (6-APA) and 4-nitrophenyl palmitate (pNPP) were purchased from Alfa Aesar Chemical Co. Ltd., China. All other chemicals and reagents were analytical grade.

Characterization:

Scanning electron microscopic (SEM) images were recorded on the FEI NanoSEM450 microscope under 5 kV accelerating voltages.

Transmission electron microscopic (TEM) images were obtained using a JEOL 2100F transmission electron microscope under 200 kV accelerating voltages.

Scanning transmission electron microscopic (STEM) images, elemental mapping and line scanning of the products were obtained using the JEOL 2100F transmission electron microscope under 4300 V accelerating voltages.

Powder X-ray diffraction (XRD) patterns were recorded using a Bruker AXS D8 Discover X-ray diffractometer with a Cu Kα anode (λ= 0.15406 nm) at 40 kV and 40 mA.

The specific surface area was calculated by the Brunauer-Emmett-Teller (BET) method. The total pore volume of the samples was estimated from the amount adsorbed at the highest P/P_0 (ca. 0.99) by the Barrett-Joyner-Halenda (BJH) model.

X-ray photoelectron spectroscopy (XPS) spectra were recorded on a Thermo Scientific K-Alpha X-ray photoelectron spectrometer.

The rheological measurements were carried out on a TA AR2000ex rheometer with a parallel plate system for samples. The frequency was fixed at 0.1 Hz and stress range was ensured in a linear viscoelastic region.

Activity Assay:

To measure BCL activity: one unit of enzyme activity was defined as the amount of BCL required to hydrolyze 1 μmol pNPP per min at 37 °C. Typically, a certain amount of free or immobilized BCL was immersed in phosphate-buffered solution (PBS, 0.1 M, pH 7.0), and the reaction was initiated by addition of 5 $mg\cdot mL^{-1}$ pNPP at 37 °C for 30 s under shaking conditions. After 3 min, the final concentrations of the products were determined by measuring the absorbance at 410 nm with an Evolution 300 ultraviolet-visible spectrophotometer.[1]

To measure PGA activity: one unit of enzyme activity was defined as the amount of PGA required to produce 1 μmol 6-APA per min at 37 °C. Typically, 1 mL of penicillin G potassium salt (4%, w/v) and a certain amount of free or immobilized PGA was mixed and

then immersed in PBS (pH 7.0) in a tube under stirring conditions (600 r/min) at 37 °C. PDAB was dissolved in methanol (0.25%, w/v) as the indicator solution for this reaction. After 10 min, 1.0 mL of the reaction sample was added to a mixture of 1.0 mL PDAB solution and 3.0 mL sodium acetate buffer (0.1 M, pH 2.5). After reacting for 10 min at room temperature, the absorbance at 415 nm was measured by using a UV spectrophotometer. The quantity of 6-APA remaining was calculated according to the standard curve.[2]

All experiments were performed in duplicates and repeated three or four times and the results from the obtained parallel data were averaged.

Preparation of nanocomposites

Preparation of BCL@H-ZIF-8: NaDC was dissolved in PBS (0.1 M, pH 7.0) to a final concentration of 0.1499 mM. Under gentle stirring, 9 mL of the NaDC solution was added to a three-neck flask at 25 °C. Then, 1 mL of a certain concentration of BCL and $Zn(NO_3)$ $6H_2O$ (the molar ratio of Zn: NaDC is 2:1) were added to the flask. After 30 min, 10 mL 2-MeIM (the molar ratio of 2-MeIM: Zn is 9:1) was added into the flask slowly with gentle stirring for 2 h at 25 °C. The products were separated and washed with PBS (0.1 M, pH 7.0) three times, and BCL@H-ZIF-8 was obtained.

To investigate the effect of pH, protein and Zn^{2+} concentrations on the morphology of BCL@H-ZIF-8, a range of pH (5-9), protein concentrations (0-4 mg mL^{-1}) and Zn^{2+} concentrations (0.075-0.60 mM) were investigated in the synthesis of BCL@H-ZIF-8.

Preparation of other nanocomposites: The syntheses of BCL@H-ZIF-67 and PGA@H-ZIF-8 were similar to that of BCL@H-ZIF-8. For synthesis of BCL@H-ZIF-67, $Co(NO_3)$ $6H_2O$ was used in place of $Zn(NO_3)$ $6H_2O$. For synthesis of PGA@H-ZIF-8, PGA replaced BCL. For synthesis of BCL@H-Fe-MOF, $Fe(Cl)_3$ $6H_2O$ and FA replaced $Zn(NO_3)$ $6H_2O$ and 2-MeIM, repectively.

Preparation of ZIF-8: $Zn(NO_3)$ $6H_2O$ (0.3695 g) was added to 4.0 mL of deionized water (DI water). Then, this solution was added to a solution of 2-MeIM (1.970 g) in 15 mL of DI

water, and the resulting mixture was stirred for a few minutes. All reactions were carried out at room temperature. Finally, the obtained products were collected by centrifugation, washed with excess DI water, and vacuum dried at room temperature.[3]

Preparation of BCL@ZIF-8: The 2-MeIM solution was prepared by dissolving 1.970 g of 2-MeIM in 15 mL of DI water. Then, BCL was mixed with 2-MeIM at 37 °C. $Zn(NO_3)$ $6H_2O$ (0.3695 g) was added to 4.0 mL of DI water to form a Zn^{2+} solution, which was poured into a flask under stirring conditions (480 r min^{-1}) at 25 °C. The 2-MeIM solution containing BCL was slowly added to the flask. After 30 min of reaction, the products were collected by centrifugation, washed with excess DI water, and vacuum dried at room temperature.[3-4]

Preparation of composites with different order of addition of precursors: The syntheses of composites with a different order of addition of precursors were similar to the procedure for synthesis of BCL@H-ZIF-8; only the order of addition of the precursors was changed. The optical images for each of these reactions with the precursors added in different sequences are shown in Figure S2.

Properties of free BCL and BCL@H-ZIF-8

To measure the resistance to protease hydrolysis, free BCL and BCL@H-ZIF-8 were incubated with papain, trypsin and alkaline protease for 3 h each in PBS (0.1 M, pH 7.0), and the activity was measured using the method as described in section 3.

To investigate acetonitrile tolerance, free BCL and BCL@H-ZIF-8 were incubated in 40% (v/v) acetonitrile solution, and the activity was measured using the method as described in section 3.

To test the reusability of the BCL@H-ZIF-8, the reactions were carried out repeatedly. After each run, the samples were collected and washed three times with PBS (0.1 M, pH 7.0) to remove the residual substrate and then used for the next reaction.

The apparent kinetic parameters (Vmax, Km, Kcat, Kcat/Km) of both free and immobilized BCL were measured by changing the concentration of pNPP and performing

nonlinear regression of the Michaelis-Menten equation.[5] All of the catalytic reactions were conducted at 37 °C.

To obtain the rate constant (Kobs) of the free and immobilized BCL, Kobs was measured by changing the duration of the pNPP hydrolyzed reaction from 30 to 180 s. The final concentrations of pNPP were determined by measuring the absorbance at 410 nm using the Evolution 300 ultraviolet-visible spectrophotometer.[6]

In all these experiments, the results were converted to relative activity or residual activity where the maximum activity was taken to be 100%.

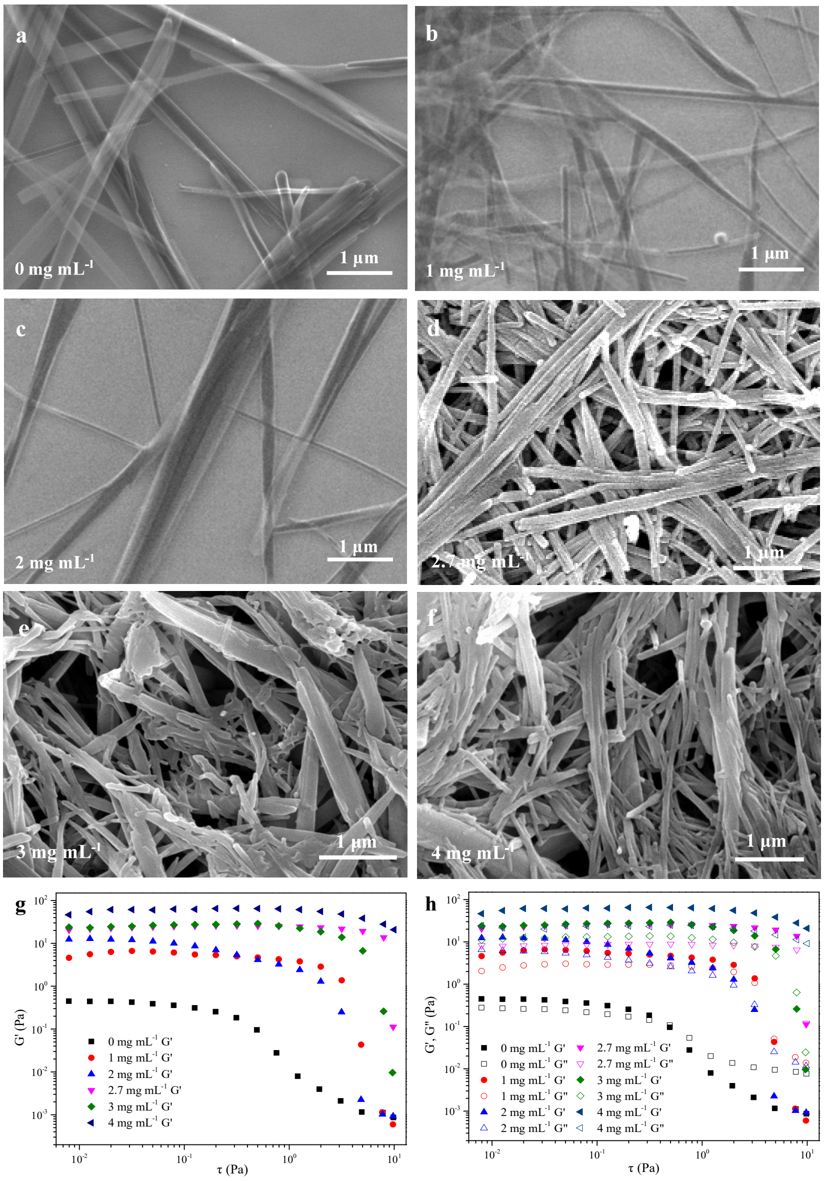

Figure S1 SEM images (a-f) and stress sweep (g-h) of hydrogel constructed under various protein concentrations

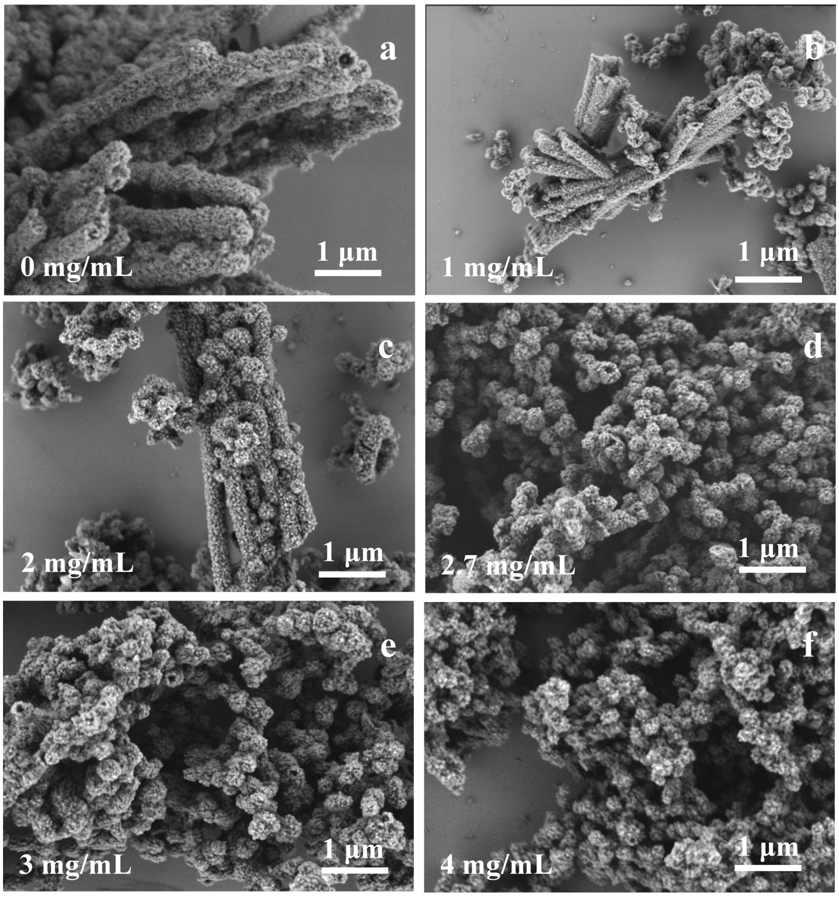

Figure S2 SEM images of BCL@H-ZIF-8 constructed under various protein concentrations

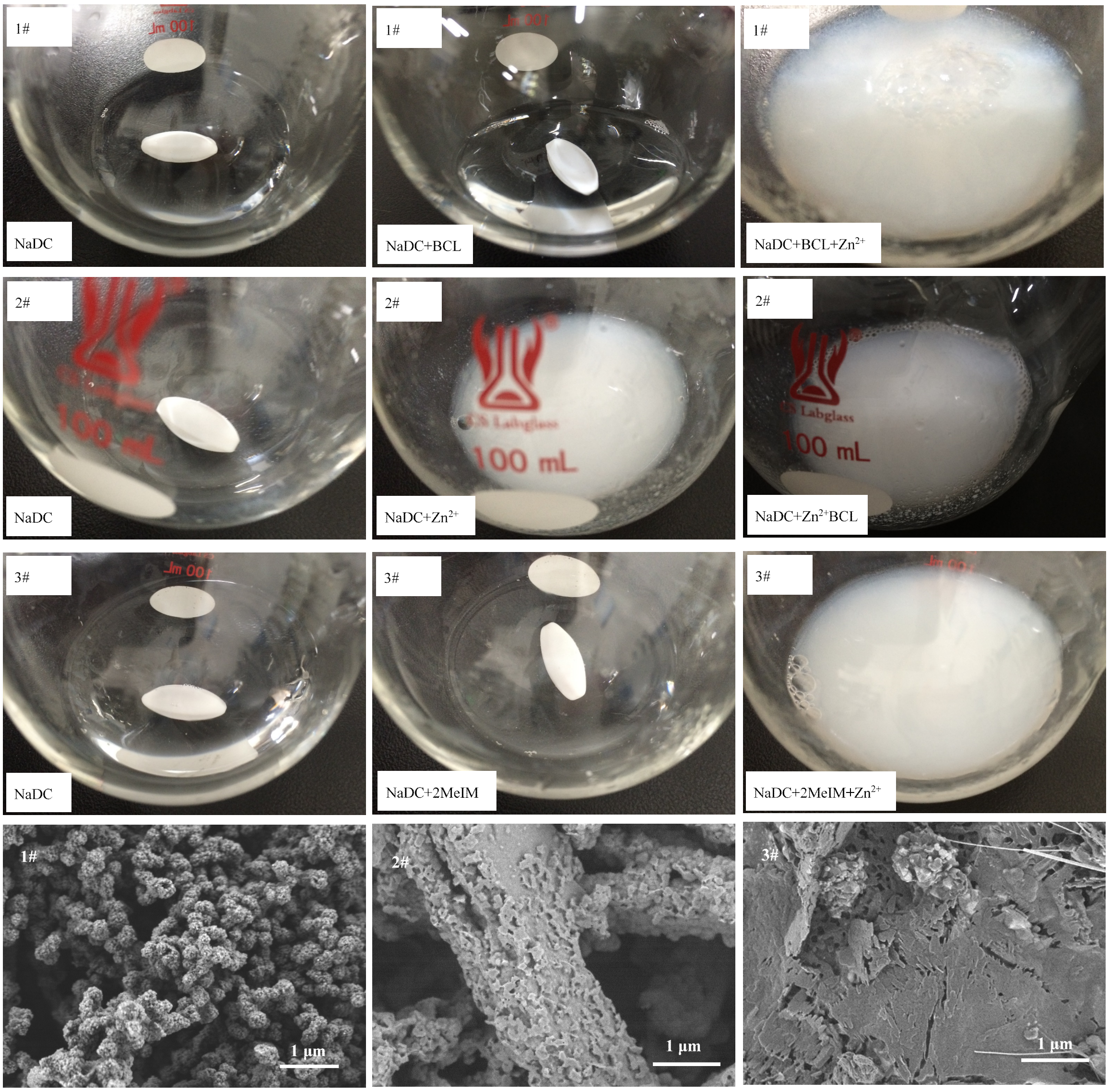

Figure S3 Optical images and the SEM images of the composites with different precursors adding order

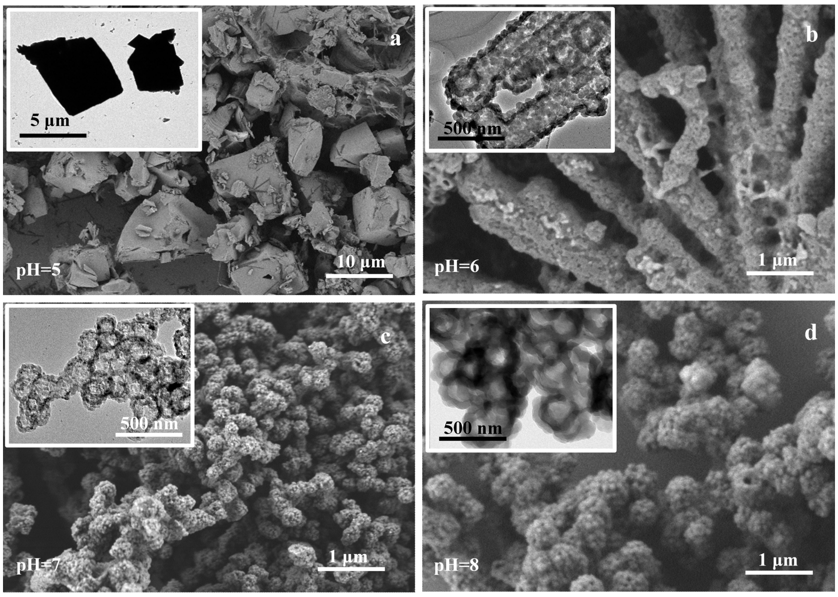

Figure S4 SEM (a-d) and TEM (a-d inset images) images of BCL@H-ZIF-8 constructed under various pH conditions

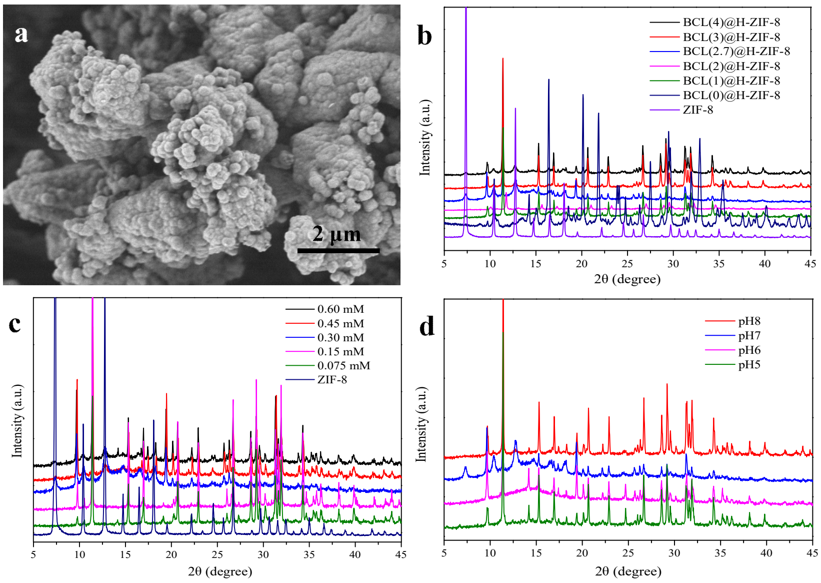

Figure S5 (a) SEM image of BCL(0)@H-ZIF-8 at pH 8, (b)XRD patterns of BCL@H-ZIF-8 constructed various conditions

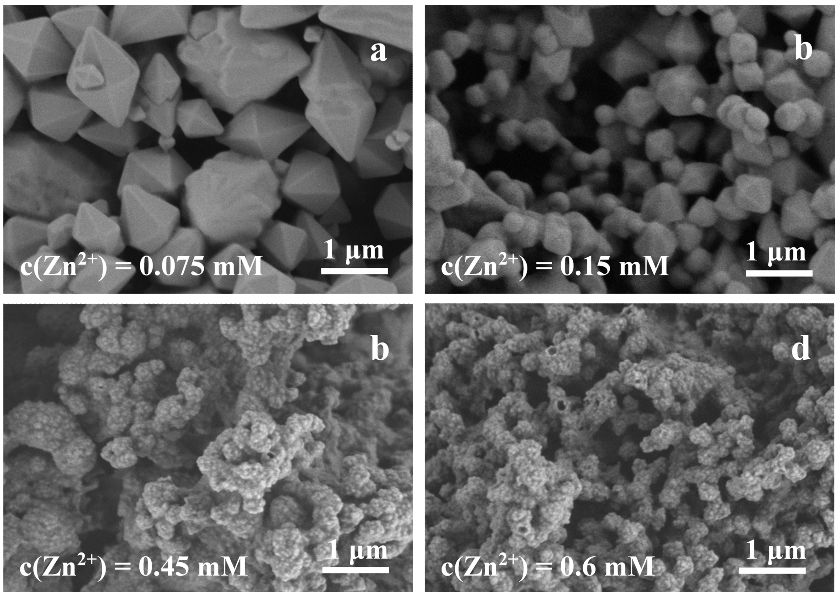

Figure S6 SEM images of BCL@H-ZIF-8 constructed under various Zn^{2+} concentrations

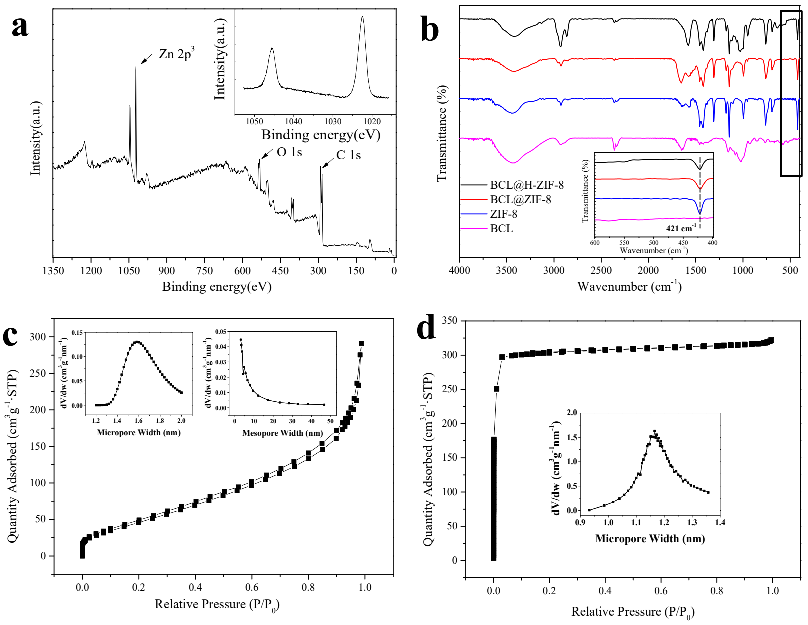

Figure S7 (a) XPS result of BCL@H-ZIF-8, (b) FT-IR spectra of different samples and (c-d) N_2 adsorption-desorption isotherms and pore-size distribution of BCL@H-ZIF-8 and pure ZIF-8

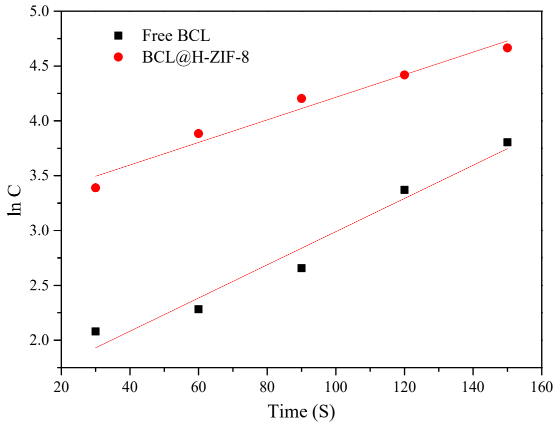

Figure S8 Kobs of free BCL and BCL@H-ZIF-8

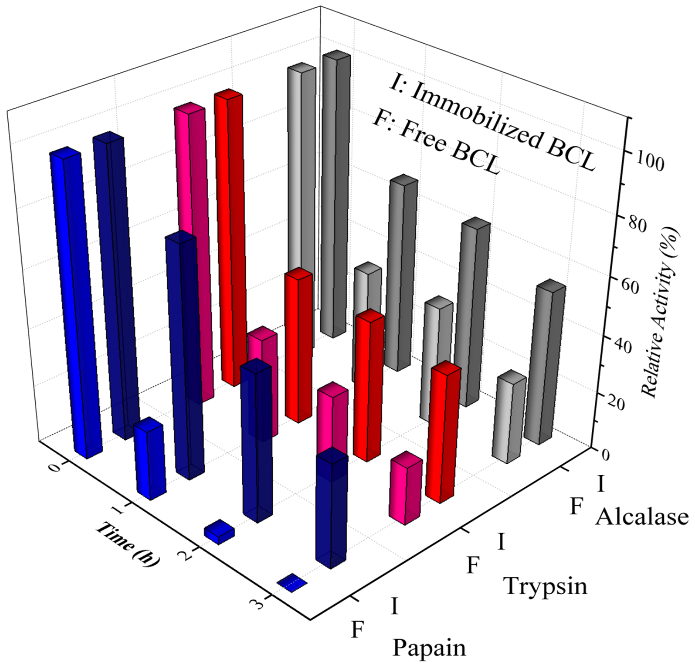

Figure S9 Relative activity of free and immobilized BCL hydrolyzed by three kinds of proteases for 3 hours

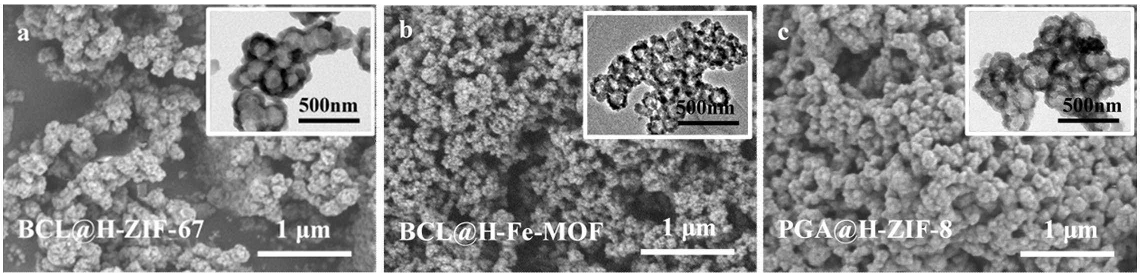

Figure S10 SEM images (a-c) and TEM images (a-c inset images) of BCL@H-ZIF-67, BCL@H-Fe-MOF and PGA@H-ZIF-8

Table S1 Kobs parameters and the enzyme activity of free BCL and BCL@H-ZIF-8

	Free BCL	BCL@H-ZIF-8
Kobs (S^{-1})	0.01513	0.01029
Enzyme activity (U g^{-1} protein)	3685	2564

Table S2 Activity and the enzyme activity recovery of free and immobilization enzyme

	Free enzyme activity ($U\ g^{-1}{}_{protein}$)	Immobilized enzyme activity ($U\ g^{-1}{}_{protein}$)	Enzyme activity recovery (%)
BCL@ZIF-8	3685	322.4	8.749
BCL@H-ZIF-8	3685	2564	69.58
BCL@H-Fe-MOF	3685	2416	65.56
BCL@H-ZIF-67	3685	3005	81.55
PGA@H-ZIF-8	41460	24892	60.04

S-References:

[1] J. K. Poppe, R. F. Lafuente, R. C. Rodrigues, M. A. Z. Ayub, *Biotechnol. Adv.* **2015**, *33*, 511.

[2] O. Romero, J. M. Guisán, A. Illanes, L. Wilson, *J. Mol. Catal. B: Enzym.* **2012**, *74*, 224.

[3] X. Wu, J. Ge, C. Yang, M. Hou, Z. Liu, *Chem. Commun.* **2015**, *51*, 13408.

[4] a) K. Liang, R. Ricco, C. M. Doherty, M. J. Styles, S. Bell, N. Kirby, S. Mudie, D. Haylock, A. J. Hill, C. J. Doonan, P. Falcaro, *Nat. Commun.* **2015**, *6*, 7240; b) V. Lykourinou, Y. Chen, X. Wang, L. Meng, T. Hoang, L. Ming, R. L. Musselman, S. Ma, *J. Am. Chem. Soc.* **2011**, *133*, 10382.

[5] G. Kemmer, S. Keller, *Nat. Protoc.* **2010**, *5*, 267.

[6] F. Shieh, S. Wang, C. Yen, C. Wu, S. Dutta, L. Chou, J. V. Morabito, P. Hu, M. Hsu, K. C. Wu, C. Tsung, *J. Am. Chem. Soc.* **2015**, *137*, 4276.
